# Diet, mycobiome and virome: from mucosal immunity to gut–brain axis regulation

**DOI:** 10.3389/fnut.2026.1873950

**Published:** 2026-06-29

**Authors:** Alessandro Medoro, Andrea Castagnetti, Mariano Intrieri, Giovanni Scapagnini, Sergio Davinelli

**Affiliations:** 1 Department of Medicine and Health Sciences "V. Tiberio", University of Molise, Campobasso, Italy; 2 Wellmicro Srl, Bologna, Italy

**Keywords:** aryl hydrocarbon receptor, bacteriophages, brain health, dietary patterns, mucosal immunity, neuroinflammation, short-chain fatty acids, tryptophan metabolism

## Abstract

The gut microbiota plays a central role in regulating host metabolism, immune function and gut–brain axis signaling. Although bacterial communities have dominated microbiome research, the intestinal ecosystem also encompasses fungal communities and bacteriophages that can influence microbial functions and host physiology. This review examines how interactions among the mycobiome, virome (principally bacteriophages), and the bacterial microbiota shape metabolic signaling pathways relevant to gut–brain axis regulation. Fungal–bacterial and phage–bacterial interactions can remodel bacterial community function through ecological competition, biofilm formation, prophage induction and horizontal gene transfer. These multi-kingdom interactions modulate key microbial metabolites, including short-chain fatty acids, tryptophan-derived indole metabolites and bile acid intermediates, which act as major regulators of intestinal barrier integrity, immune responses and neuroimmune signaling. Disruption of these metabolic pathways may contribute to altered host signaling through receptors such as the aryl hydrocarbon receptor (AhR) and bile acid receptors, with downstream effects on intestinal inflammation and neuroimmune regulation. Diet is among the most influential determinants of this ecosystem, directly shaping microbiota bacterial metabolism, fungal growth and phage–bacteria interactions. Dietary patterns rich in fermentable fibers and bioactive compounds may promote beneficial microbial metabolic outputs, whereas Western-type diets and high sugar intake may favor ecological imbalances that disrupt microbial signaling pathways relevant to gut–brain axis regulation.

## 1 Introduction

**Figure 1:**
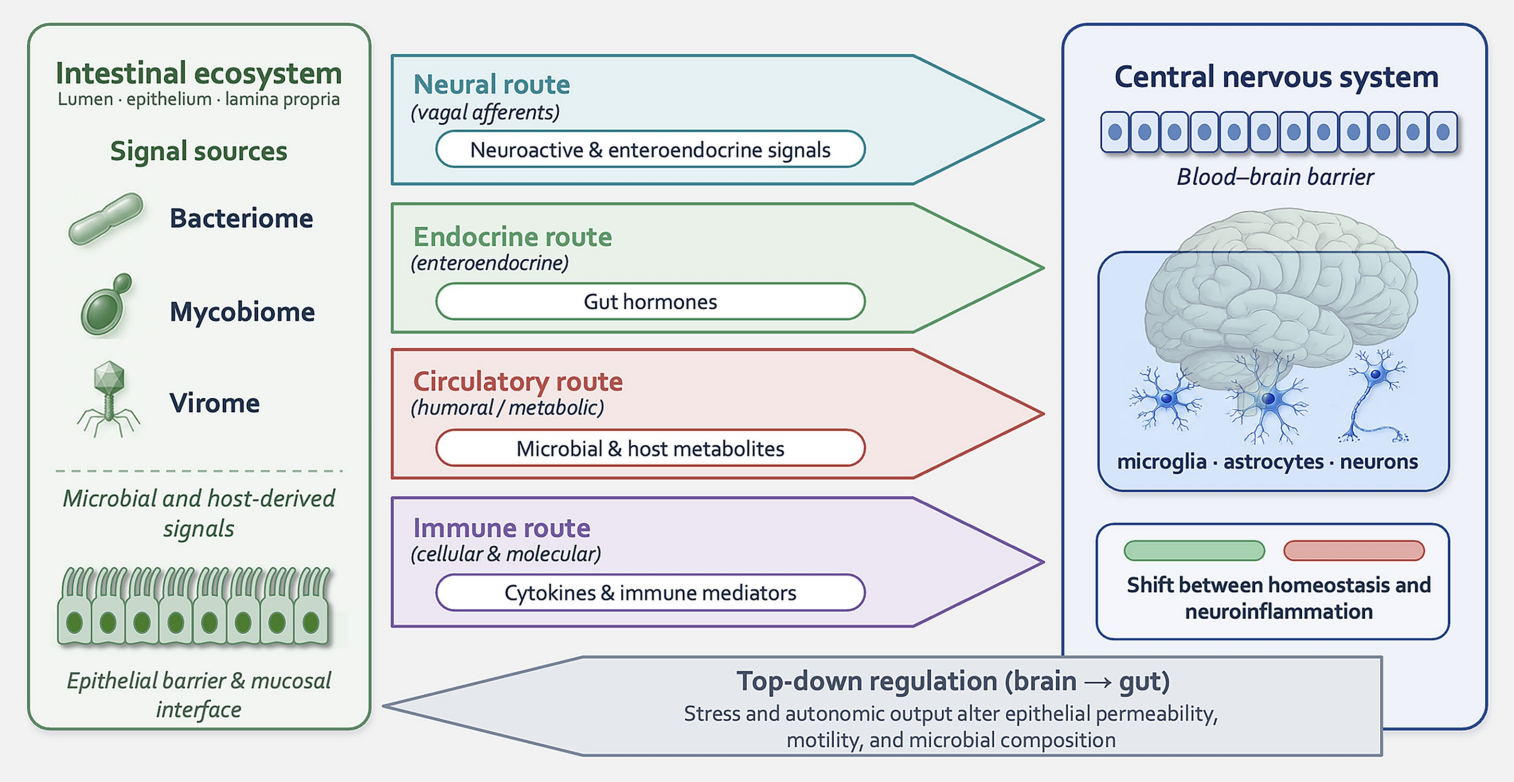
Bidirectional communication between the gut ecosystem and the CNS. Microbial and host-derived signals from the bacteriome, mycobiome, and virome reach the brain through four parallel routes, neural, endocrine, circulatory, and immune, converging on neural, glial, and vascular targets to shape the balance between homeostasis and neuroinflammation. Descending pathways close the loop, as stress and autonomic output alter intestinal permeability, motility, and microbial composition.

The gut microbiota is a dynamic, metabolically active ecosystem essential for maintaining host physiological homeostasis across the lifespan (1). Beyond its fundamental roles in nutrient processing and energy metabolism, this microbial community drives the maturation and calibration of immune responses, and the provision of colonization resistance against pathogens. These functions support continuous, bidirectional signaling with the central nervous system (CNS) through immune, metabolic, endocrine, and neural pathways, a network formally recognized as the microbiota–gut–brain axis (2, 3). The gut and the central nervous system communicate through anatomically and biochemically distinct routes that operate in parallel. Vagal afferents detect luminal microbial metabolites and relay this information to the brainstem, while enteroendocrine cells transduce the same signals hormonally through glucagon-like peptide 1 and peptide YY. Enterochromaffin cells contribute a serotonergic component, synthesizing the majority of host serotonin under microbial control. A metabolic arm runs alongside these neural and endocrine channels. Short-chain fatty acids (SCFAs), tryptophan-derived indole metabolites, and secondary bile acids cross or signal across the intestinal barrier and reach immune and neural targets, where they modulate microglial maturation, astrocyte function, and blood–brain barrier integrity. Immune mediators complete the network, since cytokines generated at the mucosal interface influence neuroinflammatory tone. These channels do not act in isolation, and their integration determines how perturbations in the intestinal ecosystem propagate to the brain. Communication is bidirectional, and descending neural and endocrine signals driven by stress and autonomic activity feedback on the gut, altering epithelial permeability, motility, and microbial composition (Figure 1) (4).

Although this microbiota–gut–brain axis paradigm has substantially advanced our understanding of host–microbe interactions, it has historically been dominated by a “bacteria-centric” view, focusing almost exclusively on bacterial taxa and their canonical metabolites (2, 3, 5). Emerging evidence, however, reveals that this view is a reductionist perspective. Non-bacterial components, specifically fungi and viruses, have emerged as potent biological modifiers of host physiology rather than mere transient colonizers. These microbial kingdoms also drive ecological interactions, immune programming, and metabolic output in ways that resonate systemically along the gut–brain axis (6, 7).

Fungi, or the “mycobiome,” comprise a numerically small fraction of the gut microbiota, representing approximately 0.1% of total microbial biomass (8). Despite this low abundance, their biological impact is profound and often disproportionate to their quantity. This functional potency arises from the strategic spatial organization and unique biochemical profile of the fungal community. The mycobiome is not uniformly distributed along the gastrointestinal tract, and like the bacterial community it varies in biomass and composition between the small and large intestine. The lower gastrointestinal tract shows a more stable and diverse fungal presence, and a limited subset of species seems to display the capacity for colonization within the intestinal niche, including *Candida*, *Saccharomyces*, *Aspergillus*, *Cryptococcus*, *Malassezia*, *Cladosporium*, *Galactomyces*, and *Trichosporon* (9).

A persistent methodological challenge in mycobiome research is distinguishing between fungal species capable of stable intestinal colonization and those transiently detected as dietary contaminants (9). This distinction has direct functional relevance: only resident fungi engage in sustained interactions with the mucosal immune system, whereas transient dietary fungi are rapidly cleared and do not establish stable colonization (9, 10). Resident fungal community is subject to continuous immunosurveillance by the host immune system through a dedicated repertoire of pattern recognition receptors (PRRs) that sense conserved cell wall components as persistent pathogen-associated molecular patterns (PAMPs). Under homeostatic conditions, this continuous immune engagement calibrates mucosal and systemic immunity without triggering inflammation (11–13). However, under conditions of metabolic stress, aging, or dysbiosis, these same persistent signals shift their functional output toward sustained inflammatory amplification. In this context, the mycobiome acts as a context-dependent amplifier of pre-existing immune and metabolic biases along the bidirectional gut–brain axis (14).

Beyond host–fungal interactions, the regulation of the intestinal ecosystem also involves complex inter-kingdom dynamics. Bacterial–fungal interactions are critically modulated by competition for shared metabolic substrates, where shifts in bacterial metabolic flux can redirect substrate utilization across kingdoms. When bacterial fermentative activity is preserved, luminal substrates are channeled toward bacterial production of SCFAs and toward the conversion of tryptophan into indole derivatives, reducing their availability to fungi. These metabolites exert a dual protective effect by suppressing fungal growth and virulence phenotypes, while concurrently supporting epithelial barrier integrity and immunoregulatory signaling (15–17). Conversely, sustained mucosa-associated fungal persistence may disrupt this balance through localized substrate competition and reducing bacterial functional output (15). These shifts establish the ecological and metabolic conditions that favor fungal persistence and downstream changes in gut-derived signaling pathways (15–20).

In parallel, the intestinal virome, predominantly composed of bacteriophages, constitutes an additional, largely underappreciated regulatory layer within the gut ecosystem. Phages modulate microbial ecology not only through lytic predation and lysogenic integration but also by reshaping bacterial metabolic programs and stress responses via horizontal gene transfer (21). This viral activity significantly influences the production of key bacteria-derived metabolites, including SCFAs, bile acid derivatives, and tryptophan-signaling molecules, thereby modulating immunity and gut–brain axis communication independently of bacterial taxonomic composition (22, 23).

Under homeostatic conditions, these multi-kingdom communities maintain a dynamic equilibrium with the host, characterized by controlled immune recognition and functional integration. However, the ecological balance of the gut ecosystem is highly sensitive to environmental stress and host-related factors, among which diet represents a dominant and continuous modulatory force (24). Dietary inputs influence the gut ecosystem at multiple levels, shaping not only bacterial communities but also fungal and viral components of the microbiota, with consequences for interkingdom interactions relevant to gut–brain communication (25–28). Many dietary patterns have been shown to influence gut fungal burden and composition, with potential downstream effects on bacteria metabolism, peripheral and central immune activation and barrier regulation (10, 25). Diet also modulates the intestinal virome primarily through indirect effects on bacterial physiology. By influencing bacterial metabolism, redox balance, and stress signaling, dietary inputs can alter phage replication dynamics and prophage induction (25, 29, 30). These virome-mediated effects may lead to functional changes in microbial metabolic output that are not apparent from bacterial taxonomic composition alone, contributing to diet-dependent variability in bacteria-derived signaling pathways, including those involving SCFAs, bile acid derivatives, and tryptophan metabolism, which are central to gut–brain axis communication (31–33).

The bacterial microbiota provides the metabolic foundation of this communication and remains the most extensively characterized component of the intestinal ecosystem. Bacterial fermentation generates the short-chain fatty acids and tryptophan-derived ligands that engage host immune and neural pathways, and bacterial enzymes drive the biotransformation of bile acids into signaling-competent species. The mycobiome and the virome operate against this bacterial background rather than independently of it, since fungal communities and bacteriophages act largely by reshaping bacterial composition and metabolic output. Interpreting their contribution to gut-brain regulation therefore requires the bacterial compartment as the reference layer, a perspective that frames the multi-kingdom analysis developed in the following sections (34).

In this review, we propose a multi-kingdom framework for the microbiota–gut–brain axis that integrates bacterial, fungal, and viral contributions to immune regulation, metabolic signaling, and CNS function. Moving beyond a bacteria-centric paradigm, we examine how these interkingdom interactions shape mucosal immunity, metabolite availability, and neuroimmune communication, and how diet emerges as a continuous and modifiable determinant of interkingdom interactions along the microbiota–gut–brain axis.

## 2 Technical challenges for non-bacterial components

Despite the increasing recognition of fungi and viruses as integral components of the gut ecosystem, their comprehensive characterization remains considerably more challenging than that of bacterial communities. These challenges arise from a combination of biological features and methodological limitations that affect sampling, sequencing strategies, and downstream bioinformatic analyses. Sample type itself shapes these interpretations. Gut microbiome studies are no longer restricted to stool, and tissue-associated approaches such as mucosal lavage or biopsies can better represent the communities that interact directly with host tissues, since fecal samples only partially reflect mucosa-associated populations (35). From a methodological perspective, both amplicon-based and shotgun metagenomic approaches present specific limitations when applied to non-bacterial components of the microbiota. In the case of the mycobiome, amplicon sequencing typically targets internal transcribed spacer (ITS) regions, which, unlike the bacterial 16S rRNA gene, lack universally conserved primer sets and exhibit substantial variability in copy number and length. This results in amplification biases and limited comparability across studies. Species-level resolution with ITS is group-dependent, reliable for many fungal taxa but limited in certain lineages, rather than uniformly reduced. Moreover, ITS-based approaches are inherently limited to taxonomic profiling and do not provide direct insights into functional potential (8, 9, 36).

Shotgun metagenomics offers a broader, untargeted alternative by enabling simultaneous detection of bacterial, fungal, and viral DNA and allowing inference of functional capacity. However, its application to the mycobiome remains constrained by the relatively low abundance of fungal DNA, the larger and more complex structure of fungal genomes, and the limited representation of fungal sequences in reference databases (10, 36). These factors lead to low mapping rates and a substantial proportion of unclassified reads, thereby limiting both taxonomic and functional interpretation. Contamination compounds these limitations. DNA from extraction kits, reagents, and the laboratory environment is a recognized source of bias in microbiome studies, and its impact is greatest for low-biomass samples, which makes fungal and viral analyses particularly vulnerable (37). Importantly, neither amplicon nor shotgun approaches can reliably distinguish between resident fungi and transient taxa introduced through diet or environmental exposure, which remains a major interpretative challenge in nutritional studies (9, 38). This distinction is particularly critical in the context of diet–microbiota interactions, where the detection of fungal DNA may reflect recent dietary exposure rather than stable ecological integration within the gut. Consequently, caution is required when interpreting shifts in fungal composition as functionally relevant, since transient fungi may not establish persistent interactions with the host or the resident microbial community (9, 36, 38).

The analytical challenges are even more pronounced for the intestinal virome. Amplicon-based strategies are not applicable to viruses due to the absence of universal phylogenetic marker genes. Virome characterization relies almost exclusively on shotgun metagenomics coupled with computational pipelines for viral sequence identification. While this approach enables the detection of both free viral particles and prophages embedded within bacterial genomes, it is highly dependent on reference databases that remain incomplete and biased toward well-characterized viral taxa (21, 39). As a result, a large fraction of viral sequences, commonly referred to as “viral dark matter”, cannot be taxonomically assigned (39, 40).

In addition to these limitations, shotgun-based virome analysis must account for the dynamic nature of bacteriophage life cycles. The coexistence of lytic and lysogenic states, together with prophage integration into bacterial genomes, complicates the attribution of sequencing reads and the interpretation of ecological and functional signals (21, 23). Notably, phage-mediated modulation of bacterial metabolism may occur in the absence of detectable changes in bacterial taxonomic composition, highlighting a key limitation of compositional microbiome analyses (22, 23).

Pre-analytical and experimental variables further contribute to variability across studies. DNA extraction efficiency, ITS primer selection, viral particle enrichment protocols, and sequencing depth can all significantly influence the detection and relative abundance of fungal and viral communities. These technical factors, combined with the intrinsic biological variability of these microbial kingdoms, currently limit reproducibility and cross-study comparability (25, 29, 38).

These considerations underscore the need for integrated and standardized analytical strategies. The combination of shotgun metagenomics with complementary approaches, such as metatranscriptomics and metabolomics, together with the development of more comprehensive reference databases and kingdom-specific bioinformatic pipelines, will be essential to improve the resolution of mycobiome and virome contributions within diet–microbiota–host interactions. These methodological constraints should be carefully considered when interpreting diet–microbiota associations, as observed changes in fungal and viral profiles may reflect technical biases, transient dietary exposure, or shifts in functional activity that are not fully captured by current sequencing approaches (41, 42).

## 3 Mycobiome in mucosal immunity and neuroinflammation

**Figure 2:**
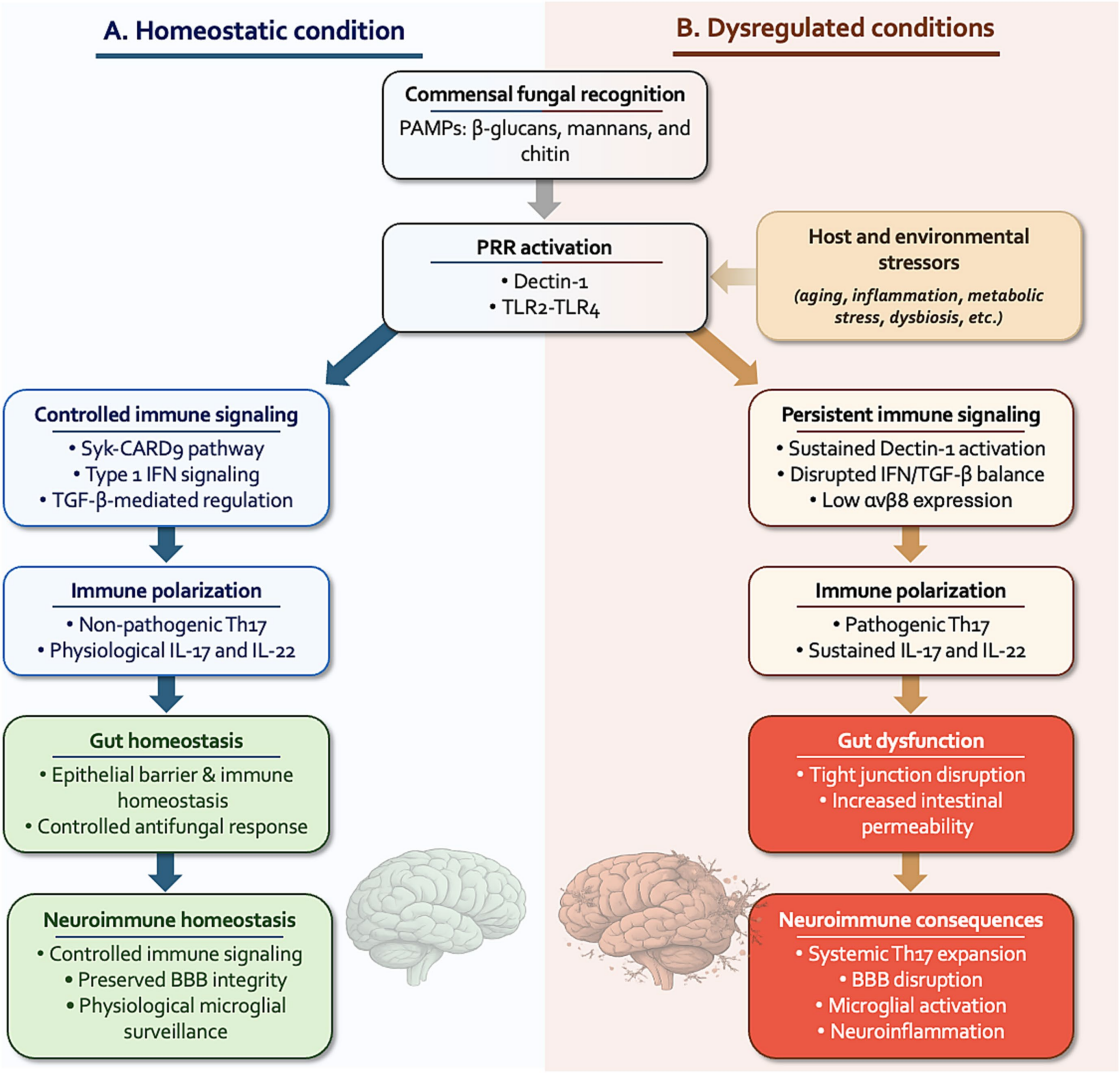
Context-dependent immune responses to fungal cell wall recognition. Commensal fungi expose cell wall PAMPs, including *β*-glucans and mannans, detected by PRRs, principally Dectin-1 and Toll-like receptors (TLR2 and TLR4). Under homeostatic conditions, PRR engagement activates controlled immune signaling involving the Syk–CARD9 pathway, type I interferon signaling, and TGF-β–mediated regulation, promoting non-pathogenic Th17 responses that support epithelial barrier integrity and intestinal immune homeostasis. In contrast, host and environmental stressors, including aging, inflammation, metabolic stress, or dysbiosis, sustain PRR signaling and disrupt regulatory pathways, leading to pathogenic Th17 polarization, intestinal barrier dysfunction, and systemic immune activation. These changes propagate along the gut–brain axis, contributing to BBB disruption, microglial activation, and neuroinflammatory processes. BBB, blood–brain barrier; CARD9, caspase recruitment domain–containing protein 9; IFN, interferon; IL, interleukin; PAMPs, pathogen-associated molecular patterns; PRR, pattern recognition receptor; Syk, spleen tyrosine kinase; TGF-β, transforming growth factor beta; TLR, Toll-like receptor; Th17, T helper 17 cells.

The functional impact of the mycobiome depends on the spatial and temporal persistence of resident fungi at the mucosal interface. While transient fungal species are rapidly cleared and many bacterial signals fluctuate with dietary inputs and microbial turnover, resident communities establish a continuous presence that subjects the host to constant immunosurveillance (Figure 2) (14). This sustained engagement shapes immune programming even in the absence of invasion and is mediated by a specialized repertoire of PRRs that discriminate fungal cell wall components from bacterial surfaces (43). Specifically, commensal and opportunistic fungi, including *Saccharomyces cerevisiae* and *Candida albicans*, express conserved cell wall components that act as PAMPs, such as *β*-glucans, mannans, and chitin. These fungal ligands are detected primarily by C-type lectin receptors, most notably Dectin-1, as well as by Toll-like receptors (TLRs), particularly TLR2 and TLR4, on myeloid cells, including dendritic cells (DCs) and macrophages. Dectin-1 engagement activates intracellular signaling via spleen tyrosine kinase (Syk) and the adaptor molecule caspase recruitment domain-containing protein 9 (CARD9), leading to nuclear factor κB (NF-κB) activation and induction of innate cytokines (11, 12, 14, 44).

A key consequence of this chronic recognition is the polarization of T helper 17 (Th17), a subset of CD4^+^ T cells specialized in mucosal defense. The Dectin-1–CARD9 signaling axis directly promotes Th17 immunity in response to commensal fungi. However, the qualitative nature of the Th17 response induced by fungal sensing is not uniform and depends on the immunological context in which pattern recognition occurs (45). Dectin-1 stimulation in human DC induces a tightly regulated type I interferon (IFN) response, characterized by transient and intermediate IFN-*β* expression. This response is controlled by the opposing activities of IFN regulatory factor (IRF)1 and IRF5 and promotes integrin αv*β*8 expression and the indirect activation of transforming growth factor (TGF)-*β*. Active TGF-β, in turn, is required for the differentiation of non-pathogenic Th17 cells, whereas dysregulated or excessive IFN-*β* signaling, associated with impaired TGF-β activation, may favor the development of pathogenic Th17 phenotypes (46). Under homeostatic conditions, balanced type I IFN responses and preserved TGF-β activation enable Th17 cells to support mucosal homeostasis without driving systemic inflammation (46). In this context, Th17-associated interleukin (IL)-17 and IL-22 contribute physiologically to barrier maintenance and epithelial repair following transient injury or infection, however, their effects are highly dependent on their duration, intensity, and inflammatory context (14, 47).

The influence of the gut mycobiome becomes most apparent under conditions of chronic immune activation, stress exposure, and aging, where subtle ecological imbalances amplify into sustained gut–brain axis dysregulation. Rather than acting as primary inflammatory triggers, mycobiome perturbations appear to stabilize and amplify pre-existing immune and metabolic biases, promoting low-grade inflammation and increased neuroimmune sensitivity (48–50). Under these conditions, sustained Th17 activity exerts maladaptive effects on barrier physiology. Chronic IL-17 exposure disrupts tight junction complexes, including claudin-1 and zonula occludens-1 expression, increasing paracellular permeability (51). In parallel, prolonged IL-22 signaling outside acute repair contexts contributes to barrier instability and increased intestinal epithelial permeability by upregulating claudin-2 expression through the Janus kinase (JAK)/Signal transducer and activator of transcription (STAT) pathway (52).

Peripheral Th17-associated immunity can propagate inflammation to the CNS. Intestinal colonization with *Candida albicans* has been shown to drive systemic expansion of fungal-specific Th17 cells and to increase IL-17 responsiveness in circulating leukocytes, shifting the baseline inflammatory set point beyond the gut compartment (43). Moreover, Th17 cells have been shown to interact with brain endothelial cells, promote blood–brain barrier (BBB) disruption through IL-17 and IL-22-dependent mechanisms, and infiltrate the CNS under persistent inflammatory conditions (53). Within the brain, IL-17 signaling exerts direct effects, where microglia express IL-17 receptors. Exposure to IL-17 activates pro-inflammatory pathways, including NF-κB, leading to increased production of cytokines and chemokines that sustain neuroinflammatory cascades (54). Experimental models of neurodegeneration confirm that Th17-associated cytokines are functional drivers, not merely correlates, of neuroinflammation and neuronal cell death (55, 56).

These observations position the mycobiome as a biologically modulator of the neuroimmune axis, able to set the inflammatory tone of the gut–brain interface rather than act as a direct pathogen. Its capacity to recalibrate Th17 activity and barrier integrity makes fungal sensing an attractive target for interventions aimed at limiting neuroinflammation.

## 4 Mycobiome and bacterial interactions in gut-brain axis regulation

Bidirectional interactions between bacterial and fungal communities are important modulators of gut–brain axis signaling. These interactions influence microbial phenotype, metabolic pathways, and immune-driven ecological selection, with consequences extending to CNS physiology and inflammatory status (57). Interkingdom balance is maintained less by stable relative abundance than by functional regulation of microbial phenotype, particularly through bacterial control of fungal morphology and metabolic activity. Commensal bacteria exert continuous suppressive pressure on fungal physiology, restraining morphogenetic programs, limiting biofilm formation, and constraining fungal persistence at the mucosal interface (38, 57).

Bacteria limit fungal behavior principally through metabolite production. SCFAs, particularly butyrate and propionate, together with bacterial tryptophan-derived metabolites suppress growth and virulence-associated phenotypes of *Candida* species, including the yeast-to-hypha transition and biofilm formation. SCFA-mediated suppression of filamentation involves transcriptional remodeling of fungal morphogenetic pathways and epigenetic regulation. This effect is consistent with the activity of SCFAs as histone deacetylase inhibitors (15, 58–60). This effect is functionally relevant because hyphal growth and biofilm formation enhance mucosal persistence, promote epithelial interaction, and amplify immune recognition through increased fungal PAMP exposure (61).

Beyond their direct antifungal effects, bacterial tryptophan-derived metabolites, including indole-3-propionic acid and indole-3-aldehyde, act as endogenous ligands of the aryl hydrocarbon receptor (AhR), a transcription factor expressed by intestinal epithelial and immune cells (62). AhR activation of signaling modulates CD4^+^ T cell differentiation by restraining pro-inflammatory T helper programs (Th1 and Th17), while promoting regulatory and barrier-supportive immune responses and favoring IL-22 production, which contributes to epithelial homeostasis and limits excessive intestinal (and peripheral) inflammation (63–65).

**Figure 3:**
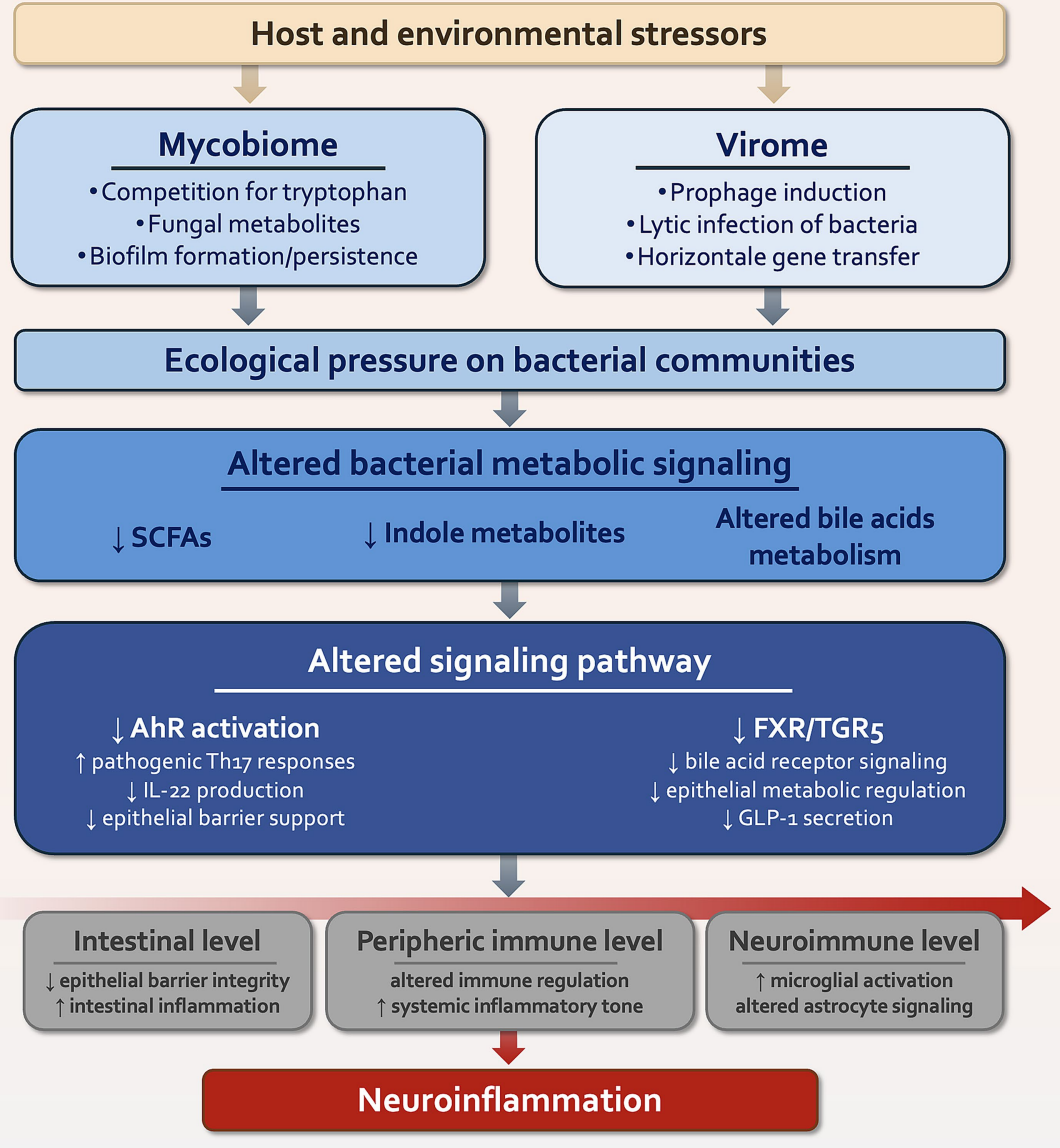
Mycobiome and virome modulation of bacterial metabolic signaling along the gut-brain axis under host and environmental stress. Bidirectional interactions between fungal communities and bacteriophages reshape bacterial metabolic functions in the intestinal ecosystem. The mycobiome influences bacterial physiology through substrate competition, fungal metabolite production, and biofilm formation, whereas the virome modulates bacterial population dynamics and transcriptional programs through prophage induction, lytic infection, and horizontal gene transfer. These multi-kingdom interactions alter key bacterial metabolic outputs, including SCFAs, indole metabolites derived from tryptophan metabolism, and bile acid intermediates, affecting host signaling through AhR and bile acid receptors (FXR and TGR5). Disruption of these regulatory pathways contributes to intestinal barrier dysfunction, altered systemic immune regulation, and neuroimmune alterations, including microglial activation and pathogenic astrocyte responses, ultimately increasing susceptibility to neuroinflammation along the gut–brain axis. AhR, aryl hydrocarbon receptor; FXR, farnesoid X receptor; GLP-1, glucagon-like peptide-1; SCFAs, short-chain fatty acids; TGR5, Takeda G protein-coupled receptor 5; Th17, T helper 17 cells.

Host and environmental stressors may impair bacterial control of fungal species, growth and morphology. Under these conditions, sustained fungal interaction with the epithelial compartment and immune system are accompanied by coordinated changes in fungal metabolic activity. These alterations initiate downstream functional, immune and metabolic effects that may propagate along the gut–brain axis (Figure 3) (17). A concrete example of this interkingdom dysregulation is found in Crohn’s disease, where *Candida tropicalis* correlates with *Escherichia coli* and *Serratia marcescens* and assembles with them into robust mixed biofilms more substantial than any single-species structure (66). Recent longitudinal profiling of patients before and after intestinal surgery has confirmed numerous bacterial–fungal correlations that track with disease state (67). These observations suggest that interkingdom biofilms could be targeted therapeutically and that paired bacterial–fungal signatures may serve as markers of disease activity, hypotheses that remain to be tested in controlled clinical studies.

Competition for substrates, particularly for aromatic compounds, emerges as a critical mechanism through which fungal–bacterial interactions may reshape gut–brain axis signaling (16, 17). Tryptophan is a central metabolic node at the interface between microbial ecology and host neuroimmune regulation: bacterial tryptophan metabolism generates indole derivatives that activate AhR, supporting epithelial integrity, immune regulation, and glial homeostasis (31).

Depending on species and growth conditions, fungal communities can compete for tryptophan and redirect its utilization toward non-canonical metabolites, such as tryptophol and phenylethanol. This process reduces the availability of bacterial-derived indole metabolites, promotes biofilm formation and interferes with bacterial quorum sensing (18–20). This diversion is amplified under sustained mucosal immune activation, where fungal persistence and spatial proximity to the epithelium facilitate localized substrate capture (18, 31). Fungal competition for substrates may contract the bacterial taxa responsible for SCFA production, while reduced indole availability weakens AhR-mediated regulatory signaling (68, 69).

Depletion of bacterial indole metabolites may have direct molecular consequences on glial signaling networks that regulate neuroinflammation (62, 70, 71). In astrocytes, AhR activation by microbiota-derived indole metabolites, in cooperation with type I IFN signaling, interferes with NF-κB–dependent transcriptional programs and suppresses astrocyte-driven inflammatory amplification. Reduced AhR ligand availability weakens this axis, favoring persistent NF-κB activation and impaired resolution of central inflammation (72). In microglia, AhR signaling regulates the balance between TGF-*α* and vascular endothelial growth factor-B (VEGF-B) production, two mediators with opposing effects on astrocyte behavior. Adequate AhR activation drives microglial TGF-α release, promoting neuroprotective, homeostatic transcriptional programs. In contrast, loss of microbial AhR ligands shifts microglial output toward VEGF-B, driving pathogenic astrocyte phenotypes associated with exacerbated neuroinflammation (73).

Microbial tryptophan metabolism also shapes functional brain networks through neuroendocrine pathways. Indole and related tryptophan metabolites modulate glucagon-like peptide-1 (GLP-1) secretion from enteroendocrine L cells in a concentration- and time-dependent manner: acute physiological exposure enhances GLP-1 release, whereas prolonged exposure attenuates this response. GLP-1 signals engage vagal afferent pathways projecting to brainstem nuclei, influencing central circuits involved in appetite control, autonomic regulation, and neuroimmune integration (74, 75). In parallel, gut bacteria modulate host serotonergic signaling indirectly, promoting serotonin synthesis in enterochromaffin cells through upregulation of tryptophan hydroxylase 1 (TPH1), the rate-limiting enzyme of mucosal serotonin production, rather than converting tryptophan to serotonin directly (76). Gut microbial metabolism of tryptophan also feeds the kynurenine pathway, generating neuromodulatory intermediates including kynurenic acid, which functions as an endogenous receptors in enterochromaffin cells (EC), specialized enteroendocrine cells (77). Activation of these sensing pathways promotes Ca^2+^-dependent serotonin release from EC cells, engaging serotonin type 3 receptors (5-HT₃R) expressed on unmyelinated vagal fibers in the intestinal mucosa and modulating brain regions involved in hunger, satiety, neurotransmitter levels, and inflammation (78). Thus, reductions in bacterial tryptophan catabolism simultaneously weaken AhR-dependent immunoregulatory pathways and disrupt serotonergic and vagal signaling that sustain to CNS homeostasis (79, 80).

## 5 Virome and gut–brain axis signaling

The intestinal virome, largely composed of bacteriophages, is a biologically active component of the gut ecosystem and an emerging regulatory layer that may contribute to microbiota–gut–brain axis communication (81). Stress exposure and inflammatory conditions have been shown to alter virome composition and prophage activation, linking environmental and immune signals to phage-mediated ecosystem remodeling (Figure 3) (82). Phages influence host physiology indirectly by reshaping bacterial population dynamics and metabolic capacity through lytic infection, lysogenic integration, and horizontal gene transfer. By acting upstream of microbial metabolite production and immune engagement, phage activity can dissociate bacterial taxonomic composition from functional output, contributing to variability in gut–brain axis signaling (1, 22, 23, 83).

Consistent with this functional role, phage–bacteria interactions regulate bacterial transcriptional programs governing carbohydrate utilization, redox homeostasis, and stress adaptation. Lysogenic phages may carry auxiliary metabolic genes and can influence bacterial regulatory programs, with potential effects on bacterial production of SCFAs, indole derivatives and other tryptophan-derived molecules, as well as bile acid derivatives, although direct evidence in the gut remains limited. These functional effects reflect stable changes in bacterial metabolic programs rather than shifts in bacterial abundance (22, 23).

Expansion of lytic phages can selectively reduce key bacterial populations, including obligate anaerobes, without producing marked reductions in overall bacterial diversity (84, 85). This selective pressure can contract regulatory metabolite pools, including SCFAs and tryptophan-derived metabolites, with downstream consequences for barrier integrity and neuroimmune regulation, as discussed above. These changes also affect bacterial functions involved in bile acid deconjugation and secondary bile acid formation (32, 84). Altered bile acid profiles modify signaling through bile acid-responsive receptors, including the farnesoid X receptor (FXR) and the Takeda G protein-coupled receptor 5 (TGR5) (32, 86, 87). FXR regulates transcriptional programs involved in bile acid transport, epithelial differentiation, antimicrobial peptide expression, and intestinal inflammatory signaling. TGR5 activation triggers cyclic AMP–dependent pathways in enteroendocrine, immune, and neural cells, influencing cytokine production and neuroendocrine outputs, including GLP-1 release (86–89). Altered FXR and TGR5 signaling have been linked to changes in gut–brain communication, epithelial barrier function, and immune regulation (90, 91).

Experimental evidence supports a causal role for the gut virome in modulating immune and behavioral outcomes relevant to brain function. In a murine model of chronic psychosocial stress, stress exposure produced reproducible alterations in gut virome composition, including enrichment of specific bacteriophage taxa, particularly within the *Caudoviricetes* class. These virome changes were accompanied by altered immune cell distributions, increased circulating cytokine concentrations, and behavioral phenotypes indicative of stress susceptibility. Transplantation of fecal virome preparations from unstressed donors protected against stress-associated behavioral alterations, immune dysregulation, bacteriome remodeling, and stress-induced transcriptional changes in the amygdala, demonstrating that phage-driven ecosystem remodeling can causally influence central stress-related outcomes (82).

Phage-induced bacterial lysis increases the release of microbial surface components, nucleic acids, and extracellular vesicles that engage PRRs on epithelial and immune cells, reinforcing innate immune activation and sustaining low-grade peripheral inflammation (6, 81). This immune amplification provides a plausible mechanistic link between virome instability and microglial priming, a process implicated in stress susceptibility and neuroinflammatory vulnerability (50, 92). Observations in human cohorts provide complementary support for the relevance of virome dynamics in chronic inflammatory and metabolic conditions frequently associated with neuropsychiatric manifestations. Alterations in gut phage richness and composition have been documented in inflammatory bowel disease and related disorders, suggesting that phage-mediated remodeling of microbial function may contribute to long-term neuroimmune vulnerability (81, 84).

Virome perturbations also intersect with fungal ecology. Phage-mediated depletion of bacterial taxa that normally restrain fungal growth through metabolic and competitive mechanisms, can promote fungal persistence, biofilm formation, and increased exposure of immunostimulatory fungal cell wall components. These secondary effects may amplify mycobiome-driven immune activation, reinforcing Th17-associated programs linked to BBB disruption and susceptibility to neuroinflammation. In this context, virome perturbations may indirectly modulate fungal immunogenicity and contribute to coordinated multi-kingdom effects along the microbiota–gut–brain axis (11).

Age-related changes in the intestinal virome, frequently sustained by oxidative stress and inflammation, may also progressively alter phage–bacteria interactions with consequences for gut–brain axis signaling. Aging is associated with increased virome instability, reduced phage diversity and network resilience, and shifted lytic-to-lysogenic ratios, changes that often precede detectable shifts in bacterial taxonomy, identifying the virome as an early and sensitive layer of ecological dysregulation (21, 39, 40, 93, 94). In older individuals, expansion of lytic phage populations and increased prophage induction have been linked to heightened bacterial stress responses and reduced functional redundancy among metabolically specialized taxa. This loss of redundancy disproportionately impacts SCFA production, bile acid transformation, and tryptophan metabolism, amplifying the metabolic and functional consequences of otherwise modest compositional shifts (22, 95, 96). Concurrently, age-associated immune activation and increased epithelial permeability create conditions that favor prophage induction and phage-mediated bacterial turnover, reinforcing a feedback loop between inflammation and virome instability (36, 84). These dynamics converge with reduced barrier resilience, altered bile acid signaling, and heightened neuroimmune sensitivity characteristic of aging. Phage-driven reductions in bacterial metabolic capacity may therefore contribute to the emergence of inflammaging-associated signaling profiles and to increased interindividual variability in neuroimmune and cognitive outcomes observed in later life (92, 97).

## 6 Diet in mycobiome and virome regulation

Diet is a primary, although still incompletely characterized, driver of interindividual variability in gut mycobiome and virome colonization. The consequences of these dietary influences extend well beyond intestinal physiology, reaching into systemic immune regulation and brain function (Table 1) (27, 38).

**Table 1:** Dietary patterns influencing gut mycobiome and virome dynamics and potential implications for the gut–brain axis.

Dietary pattern	Effects on mycobiome and virome	Indirect bacterial consequences	Potential implications for the gut–brain axis
High-fiber/plant-based diets	Promotion of fermentative microbial metabolism; SCFAs-mediated inhibition of fungal hyphal growth and biofilm formation; diet-associated changes in viral populations	Increased SCFAs and indole derivatives supporting epithelial and immune signaling	*Beneficial modulation*: support of epithelial homeostasis and immunoregulatory signaling relevant to gut–brain communication
Animal-based/processed diets	Rapid changes in fecal fungal detection including food-associated taxa; persistent alterations in gut fungal communities	Altered microbial metabolic activity and reduced fermentative metabolism	*Potential disruption*: shifts in microbial signaling pathways influencing gut–brain axis regulation
High-fat diets	Virome remodeling with reduced Siphoviridae, expansion of Microviridae, and signatures of more lytic viral strategies; altered fungal community structure	Bacterial dysbiosis and altered metabolism	*Potential disruption*: altered bile acid signaling and inflammatory pathways relevant to gut–brain communication
High-sugar/refined carbohydrate/fructose-enriched diets	Promotion of Candida abundance and fungal biofilm traits; increase of prophage induction and phage production	Altered bacterial competition and reduced metabolic diversity	*Potential dysregulation*: increased mucosal immune activation and altered microbial signaling relevant to neuroinflammation
Gluten-free diet	Diet-responsive changes in virome composition influenced by baseline virome diversity and ecological buffering capacity	Variable bacterial functional consequences depending on baseline ecosystem structure	Context-dependent: modulation of microbial ecosystem dynamics relevant to gut–brain signaling

SCFAs, short-chain fatty acids.

One of the earliest systematic investigations of diet–fungal associations in humans used culture-independent sequencing to identify 66 fungal genera in fecal samples. In this cohort, *Candida*, *Cladosporium*, and *Saccharomyces* emerged as the most prevalent taxa. The relative abundance of *Saccharomyces* was hypothesized to reflect the direct consumption of yeast-containing foods, including bread and fermented beverages, whereas higher levels of *Candida* correlated strongly with recent intake of carbohydrate-rich diets (98). These findings raise an important interpretative challenge: distinguishing diet-derived fungi from species capable of stable, long-term colonization of the intestinal niche (38). Dietary modulation of the mycobiome should be interpreted in functional terms, differentiating between transient exposure with immune tolerance and changes associated with inflammatory amplification and subsequent effects on gut–brain axis signaling (38, 99).

Short-term dietary interventions demonstrate the sensitivity of the mycobiome to nutritional inputs. In a controlled feeding study, David et al. demonstrated that rapid transitions between animal-based and plant-based diets altered gut microbial community structure, including the detection of diverse fungal genera such as *Scopulariopsis*, *Penicillium*, *Debaryomyces*, and *Candida* (26). Several of these taxa are commonly associate with food production, and species such as *Penicillium* and *Debaryomyces* exhibit limited growth at physiological temperature. This suggests that their detection in fecal samples reflects passive dietary exposure rather than durable ecological integration into the gut (9). In murine models, a processed diet induces persistent alterations in gut fungal communities. These shifts correlate with increased body mass, hepatic fat deposition, transcriptional metabolic adaptations, and altered serum biomarkers, implicating the mycobiome, specifically the genera *Thermomyces* and *Saccharomyces*, as active participants in host metabolic regulation (100). Clinical data reinforce this relevance, suggesting that dietary modulation of the mycobiome carries profound pathophysiological relevance. In patients with Crohn’s disease undergoing exclusive enteral nutrition, the observed clinical improvements and normalization of inflammatory markers coincide with distinct shifts in the gut ecosystem, including a significant reduction in fungal taxa. This suggests that nutritional therapies may modulate host–microbe immune interactions through pathways that extend beyond the bacterial compartment alone. Diets that restrict fermentable substrates or selectively alter microbial competition may constrain the persistence and immunostimulatory potential of opportunistic fungi, mitigating mucosal inflammation and improving disease outcomes (101).

These effects are mechanistically mediated by diet-dependent control of bacterial ecological dominance, which serves as a primary determinant of fungal behavior in the gut. At the metabolic level, these interactions converge on SCFA- and tryptophan-regulatory pathways. Diets sustaining fermentative bacterial metabolism promote SCFA availability and bacterial conversion of tryptophan into indole derivatives, supporting AhR-dependent signaling (63, 102, 103). Conversely, dietary patterns low in fiber and enriched in saturated fatty acids or simple carbohydrates suppress fermentative metabolism and reduce SCFA production. This metabolic shift permits fungal diversion of tryptophan metabolism, reinforcing epithelial vulnerability and exacerbating immune activation along the gut–brain axis (17, 102).

The influence of diet on the intestinal virome is predominantly indirect, mediated through changes in bacterial host physiology. Because bacteriophages are obligately dependent on their bacterial hosts’ metabolic state and stress responses, dietary inputs remodel phage activity by shifting viral replication programs, altering redox balance, and modulating prophage induction. In a longitudinal intervention in healthy adults consuming defined diets with contrasting fat and fiber content, shotgun metagenomic analysis identified significant diet-associated changes in viral populations. Interestingly, a partial convergence was observed among individuals consuming similar diets, confirming the *in vivo* responsiveness of the gut virome to nutritional changes (25). A subsequent longitudinal analysis examining a gluten-free diet found that the magnitude of virome compositional changes was contingent upon baseline diversity. This aligns with the concept of ecological “buffering capacity,” whereby the initial state of the ecosystem conditions its resilience or responsiveness to dietary perturbations (29).

Mechanistic evidence links dietary composition to phageome remodeling primarily through metabolically mediated effects on bacterial hosts, with SCFAs emerging as a central integrative signal. In a longitudinal dietary study, Minot et al. demonstrated that a low-fat, high-fiber diet drove the human gut phageome toward a distinct ecological state, despite only modest changes in overall bacterial taxonomic composition (25). Mechanistic support for this causal link comes from experimental models showing that nutrient-driven changes in bacterial metabolism directly regulate prophage induction. For example, in a gut symbiont model, a fructose-enriched diet stimulated bacteriophage production from *Lactobacillus reuteri* prophages during gastrointestinal transit: enhanced carbohydrate flux increased SCFA generation, activated the acetate kinase (Ack) pathway, and engaged a RecA-dependent stress response, ultimately promoting prophage induction and viral replication (30). These findings indicate that diets favoring fermentative metabolism can modulate virome activity by altering bacterial physiological states, with potential consequences for gut–brain axis signaling (104).

Reflecting the acute sensitivity of the virome to host metabolic state, transition from standard chow to a high-fat diet in mice produced marked shifts in fecal virome *β*-diversity, characterized by a reduction in *Siphoviridae* and a concomitant expansion of *Microviridae*. These taxonomic rearrangements were accompanied by gene-content signatures indicative of a strategic shift toward more lytic viral lifecycles, evidenced by a reduced prevalence of integrase genes. This pattern is compatible with increased bacterial stress and reduced lysogenic buffering, suggesting that high-fat feeding promotes a virome configuration associated with metabolic instability (105). These diet-associated shifts in virome activity are consistent with a mechanistic framework in which bacterial stress responses, driven by inflammatory and redox changes, promote prophage induction through conserved RecA-dependent pathways. Such stress signals are plausibly generated under diet-induced inflammatory or metabolic states, providing a mechanistic bridge between nutrient availability, bacterial physiology, and phage replication dynamics (106, 107).

## 7 Conclusion

Gut–brain axis regulation emerges from the coordinated activity of multiple microbial kingdoms within the intestinal ecosystem rather than from bacterial communities alone. Fungal communities and bacteriophages constitute additional ecological layers capable of reshaping microbial interactions and bacterial metabolic activity. Through substrate competition, metabolic interference, and phage-mediated genetic exchange, these multi-kingdom interactions regulate the production of key microbial metabolites such as SCFAs, tryptophan-derived indole metabolites and bile acid intermediates. The metabolite landscape extends beyond these classes. Microbial neuroactive compounds, including *γ*-aminobutyric acid and other neurotransmitter precursors, aromatic amino acid catabolites such as p-cresol, and microbial extracellular vesicles carrying diverse bioactive cargo, are increasingly proposed as additional routes of gut–brain communication (108). Because these metabolites regulate intestinal barrier integrity, immune responses and neuroimmune signaling, alterations in multi-kingdom microbial dynamics may contribute to altered gut–brain communication.

The mycobiome exerts its greatest influence when the intestinal ecosystem is challenged by physiological or environmental stressors such as aging, metabolic imbalance or microbial dysbiosis. Under these conditions, sustained fungal persistence at the mucosal interface shifts immune signaling from a homeostatic toward a pathogenic profile. Amplification of Th17-associated responses, impairment of epithelial barrier integrity and the propagation of low-grade inflammatory signaling represent mechanisms through which fungal–bacterial interactions may contribute to neuroimmune alterations relevant to gut–brain axis regulation.

The study of the intestinal mycobiome and virome nonetheless faces significant conceptual and methodological challenges. The low biomass and marked temporal variability of fungal communities, together with the influence of dietary exposure, complicate the distinction between resident taxa and transient fungi introduced through food. Most mechanistic evidence derives from experimental models, and direct causal evidence in humans remains limited. The intestinal virome presents analogous challenges: phage-mediated modulation of bacterial metabolic capacity may occur without detectable shifts in bacterial taxonomy, complicating the interpretation of compositional microbiome data.

Within this complex ecological framework, diet likely represents the most tractable driver of multi-kingdom microbial dynamics. Nutritional patterns simultaneously influence bacterial metabolism, fungal growth and phage–bacteria interactions, thereby shaping microbial metabolite production and host signaling pathways involved in gut–brain axis regulation. Integrating dietary assessments with longitudinal and multi-omics analyses of bacterial, fungal and viral communities will be essential to clarify how interkingdom microbial dynamics contribute to gut–brain communication and to identify potential nutritional strategies capable of modulating these processes.

Several questions remain open. It is still unclear which fungal and viral taxa are stable functional members of the ecosystem rather than diet-derived passengers, and whether their metabolic contributions are quantitatively relevant beside the bacterial majority. Addressing these gaps will require methodological standards tailored to low-biomass communities, with rigorous contamination controls and mucosa-aware sampling. Defined-community and gnotobiotic models that reconstruct fungal, bacterial, and phage interactions offer a route to move from correlation to causation. Emerging directions include single-cell and spatial approaches to resolve interkingdom interactions *in situ*, and host-stratified studies in aging and inflammatory disease, where multi-kingdom dynamics are most likely to shape gut–brain outcomes.
